# Seroprevalence and Severity of 2009 Pandemic Influenza A H1N1 in Taiwan

**DOI:** 10.1371/journal.pone.0024440

**Published:** 2011-09-01

**Authors:** Chih-Jung Chen, Ping-Ing Lee, Shih-Cheng Chang, Yhu-Chering Huang, Cheng-Hsun Chiu, Yu-Chia Hsieh, Shang-Chwen Chang, Feng-Yee Chang, Jen-Jyh Lee, Shey-Chiang Su, Gwan-Han Shen, Yin-Ching Chuang, Yao-Shen Chen, Jien-Wei Liu, Tzou-Yien Lin

**Affiliations:** 1 Divisions of Pediatric Infectious Diseases, Department of Pediatrics, Chang Gung Children's Hospital and Chang Gung Memorial Hospital, Taoyuan, Taiwan; 2 Department of Pediatrics, National Taiwan University Hospital and National Taiwan University College of Medicine, Taipei, Taiwan; 3 Research Center for Emerging Viral Infections, Chang Gung University, Taoyuan, Taiwan; 4 Department of Medical Biotechnology and Laboratory Science, Chang Gung University, Taoyuan, Taiwan; 5 College of Medicine, Chang Gung University, Taoyuan, Taiwan; 6 Department of Internal Medicine, National Taiwan University Hospital, Taipei, Taiwan; 7 Center for Disease Control, Taiwan, Taipei, Taiwan; 8 Department of Internal Medicine, Buddhist Tzu Chi General Hospital, Hualien and Tzu Chi University, Hualien, Taiwan; 9 Division of Infectious Disease, Department of Internal Medicine, Mackay Memorial Hospital, Hsinchu, Taiwan; 10 Division of Respiratory and Critical Care Medicine, Department of Internal Medicine, Taichung Veterans General Hospital, Taichung, Taiwan; 11 Division of Infectious Diseases, Department of Internal Medicine, Chi-Mei Medical Center, Tainan and Liouying, Taiwan; 12 Division of Infectious Diseases and Clinical Microbiology, Kaohsiung Veterans General Hospital, Kaohsiung, Taiwan; 13 School of Medicine, National Yang-Ming University, Taipei, Taiwan; 14 Division of Infectious Disease, Department of Internal Medicine, Chang Gung University-Kaohsiung, Chang Gung University Medical College, Kaohsiung, Taiwan; University of Texas Medical Branch, United States of America

## Abstract

**Background:**

This study is to determine the seroprevalence of the pandemic influenza A H1N1 virus (pH1N1) in Taiwan before and after the 2009 pandemic, and to estimate the relative severity of pH1N1 infections among different age groups.

**Methodology/Principal Findings:**

A total of 1544 and 1558 random serum samples were collected from the general population in Taiwan in 2007 and 2010, respectively. Seropositivity was defined by a hemagglutination inhibition titer to pH1N1 (A/Taiwan/126/09) ≥1:40. The seropositivity rate of pH1N1 among the unvaccinated subjects and national surveillance data were used to compare the proportion of infections that led to severe diseases and fatalities among different age groups. The overall seroprevalence of pH1N1 was 0.91% (95% confidence interval [CI] 0.43–1.38) in 2007 and significantly increased to 29.9% (95% CI 27.6–32.2) in 2010 (p<0.0001), with the peak attack rate (55.4%) in 10–17 year-old adolescents, the lowest in elderly ≥65 years (14.1%). The overall attack rates were 20.6% (188/912) in unvaccinated subjects. Among the unvaccinated but infected populations, the estimated attack rates of severe cases per 100,000 infections were significantly higher in children aged 0–5 years (54.9 cases, odds ratio [OR] 4.23, 95% CI 3.04–5.90) and elderly ≥ 65years (22.4 cases, OR 2.76, 95% CI 1.99–3.83) compared to adolescents aged 10–17 years (13.0 cases). The overall case-fatality rate was 0.98 per 100,000 infections without a significant difference in different age groups.

**Conclusions/Significance:**

Pre-existing immunity against pH1N1 was rarely identified in Taiwanese at any age in 2007. Young children and elderly – the two most lower seroprotection groups showed the greatest vulnerability to clinical severity after the pH1N1 infections. These results imply that both age groups should have higher priority for immunization in the coming flu season.

## Introduction

**Figure 1:**
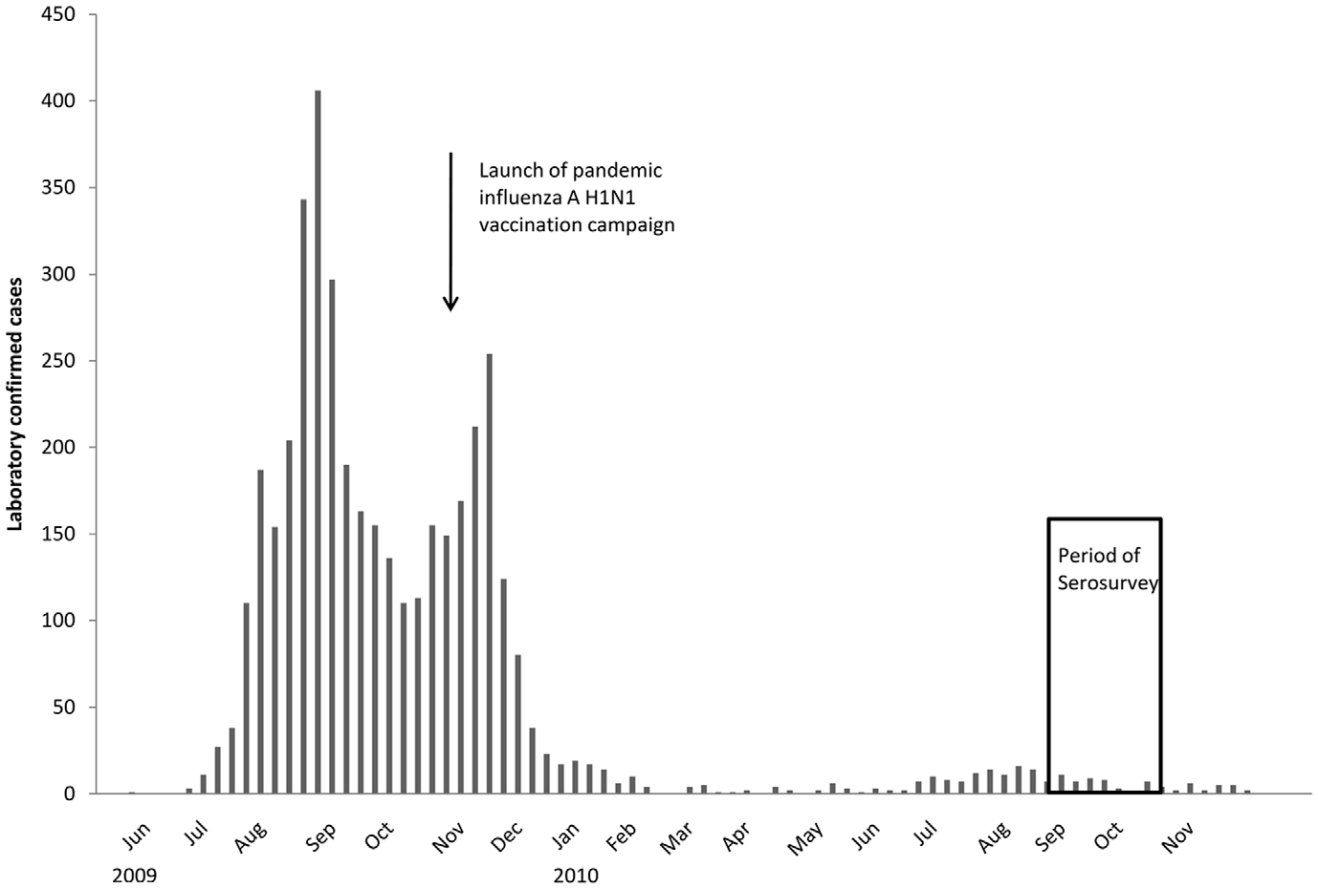
National epidemic surveillance data of 2009 pandemic influenza A H1N1 in Taiwan.

The 2009 pandemic influenza A H1N1 virus (pH1N1) was initially identified in Mexico and United States during March and April of 2009, subsequently transmitted in communities across North America within weeks, and identified in many areas of the world by May 2009 [1]–[5]. On June 11, 2009, the World Health Organization (WHO) declared a global pandemic [6]. Worldwide transmission of the pH1N1 virus continued, and most countries experienced one or two epidemic waves before the end of the pandemic [7]–[10]. In Taiwan, the first laboratory confirmed case was identified on May 20, the first severe complicated case was reported on July 17, and the first fatal case was reported on July 31. As of August 31, 2010, a total of 983 laboratory confirmed severe cases were reported to the Taiwan Centers for Diseases Control (CDC) and 50 of them died. Two major waves occurred between July and December of 2009, and only dispersed cases appeared in 2010 (Figure 1).

To better understand the population's immunity against pH1N1 in Taiwan before and after the pandemic, we conducted a seroepidemiology study 8 months after the last wave of the epidemic. The epidemiological factors associated with seropositivity were explored. Combining the seroprevalence data from the unvaccinated subjects and the national epidemiological surveillance data of severe and fatal cases, we estimated the extent of the pH1N1 infections in the community and compared the age-stratified severity of pH1N1 infections. The findings from this study should provide useful information for national public health authorities in the preparedness of the pandemic influenza in the future.

## Results

### Seroprevalence of pH1N1 in 2007 and 2010

The overall seroprevalence of pH1N1 in Taiwan estimated by hemagglutination inhibition (HI) assay was 0.91% (14 of 1544 samples) and 29.9% (466 of 1558 samples) in 2007 and 2010, respectively. The seropositivity in baseline samples before the global pandemic was not confined to the elder population, but it was scattered among subjects beyond the school ages (median 51.0 years, range 14.0–89.8 years). In the post-pandemic period, seropositivity was significantly higher among subjects <18 years old compared to subjects ≥18 years old (38.9% vs. 26.7%, respectively, p <0.0001). The age-specific distribution of seropositivity rates disclosed an increasing trend from young children <4 years old (23.3%) to a peak in adolescents at 10–17 years of age (55.4%; Table 1). The seropositivity rate ranged from 29.7% to 33.0% in adults 18–44 years of age and declined to a nadir (14.1%) in the elderly ≥65 years old.

**Table 1:** Age-specific seroprevalence and geometric means of hemagglutination inhibition titer to novel influenza A (H1N1) in Taiwan during the pre-pandemic (2007) and post-pandemic (2010) periods.

Age, yrs	HI titers to 2009 pandemic influenza A (H1N1)					
	2007 (n = 1544)		2010 (all subjects, n = 1558)		2010 (unimmunized subjects, n = 912)	
	GMT (95% CI)	% with titer ≥40 (95% CI)	GMT (95% CI)	% with titer ≥40 95% CI)	GMT (95% CI)	% with titer ≥40 (95% CI)
<2	8.8 (7.4–10.3)	0.0 (-)	64.5 (20.7–108.3)	24.2 (9.6–38.9)	77.0 (14.7–139.2)	17.4 (1.9–32.9)
2–3	7.7 (6.4–9.0)	0.0 (-)	45.7 (21.3–70.1)	22.6 (11.4–33.9)	37.6 (18.7–56.6)	23.5 (3.4–43.7)
4–5	7.4 (6.1–8.8)	0.0 (-)	35.8 (30.3–41.3)	32.1 (24.3–39.9)	36.6 (27.6–45.5)	28.4 (17.6–39.2)
6–9	8.0 (6.8–9.2)	0.0 (-)	35.0 (28.8–41.2)	42.9 (31.3–54.5)	38.3 (24.2–52.3)	47.8 (27.4–68.2)
10–17	9.8 (8.6–11.0)	1.33 (0–3.17)	52.1 (42.1–62.1)	55.4 (46.5–64.2)	52.8 (32.6–73.0)	47.7 (33.0–62.5)
18–24	7.9 (6.9–8.8)	0.63 (0–1.86)	38.0 (28.5–47.5)	29.7 (22.5–36.9)	29.2 (24.8–33.6)	22.2 (14.7–29.8)
25–34	8.9 (7.2–10.6)	1.03 (0–2.44)	32.8 (28.8–36.8)	32.9 (26.9–38.9)	26.2 (23.3–29.1)	18.9 (12.6–25.2)
35–44	8.2 (6.5–9.9)	0.51 (0–1.51)	36.7 (29.5–43.9)	33.0 (26.8–39.3)	24.5 (22.8–26.3)	20.1 (13.5–26.8)
45–54	6.4 (5.8–7.0)	0.50 (0–1.48)	33.2 (26.4–40.0)	24.9 (19.1–30.6)	26.3 (21.3–25.9)	14.6 (8.7–20.5)
55–64	7.3 (6.3–8.3)	1.90 (0–4.03)	25.9 (23.8–28.0)	20.2 (14.2–26.3)	23.8 (21.8–25.9)	15.4 (8.5–22.3)
≥65	7.7 (6.8–8.5)	1.79 (0.05–3.54)	23.2 (21.0–25.4)	14.1 (8.5–19.7)	21.9 (20.5–23.4)	11.8 (5.3–18.4)
Overall	8.0 (7.6–8.4)	0.91 (0.43–1.38)	35.2 (32.8–37.6)	29.9 (27.6–32.2)	29.5 (27.1–31.8)	20.6 (18.0–23.2)

Abbreviations: GMT, geometric mean titer; CI, confidence interval

The geometric mean titers (GMTs) of HI among each age group are displayed in Table 1. In the pediatric population the GMT was higher for subjects at the ages of <2 years (64.5±128.3) and 10–17 years (52.1±56.0) than subjects at the age of 2 to 9 years (37.6±49.1) in the post-pandemic samples. The GMT of HI among the adult populations generally followed the trend of seropositivity and declined from 38.0 in subjects aged 18–24 years to the lowest level (23.2±13.5) in the elder population ≥65 years.

**Figure 2:**
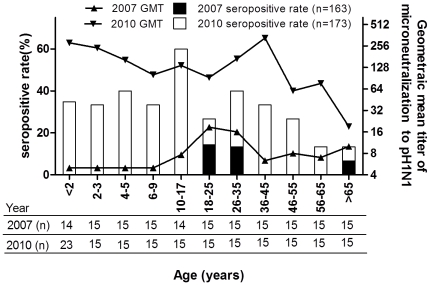
Geometric mean titers and seropositivity rates among different age groups of Taiwanese before and after 2009 pandemic, estimated by microneutralization assay.

The microneutralization (MN) titers were measured in 336 randomly selected samples and a strong correlation was identified between the MN and HI titers (Pearson correlation coefficient 0.85291, p<0.0001). A MN titer of ≥1∶80 was identified in 3.1% and 32.4% of pre-pandemic and post-pandemic samples, respectively. Figure 2 displays the age-specific distribution of seroprevalence and GMTs of MN assay, which are very similar to the patterns generated by HI assay. For instance, the seropositive rate in 2010 peaked at the age of 10–17 years and was significant higher in children <18 years old than in adults ≥18 years (39.8% vs. 25.6%, p = 0.0461). The decline of seropositive rate with age in adult population was also compatible with the results of HI assay.

### Seropositivity in unvaccinated population and the age-stratified severity of pH1N1 infections

Among the 912 subjects without immunization against pH1N1, 188 (20.6%) had an HI titer ≥1∶40. The age-specific distribution of seropositivity in unimmunized subjects was similar to the trend identified in all study subjects (Table 1), with the highest rates occurring in children 6–9 and 10–17 years of age (47.8% and 47.7%, respectively) and two significant declining trends toward the extremes of the age spectrum (p = 0.0323 for subjects <10 years old, and p<0.0001 for subjects ≥10 years old). Based on the assumption that the incidences of natural infections in the unimmunized population were proportional to the whole population, we compared the severity of infection between each age group and the three regions of study (Table 2). The greatest case-serious infection rate occurred in children 0–5 years of age (54.8–55.0 cases/100,000 infections), which was approximately four folds (odds ratio [OR] 4.23, 95% confidence interval [CI] 3.04–5.90) higher than the rate in adolescents 10–17 years of age (13 cases/100,000 infections). The elderly ≥65 years also had nearly three folds higher case-serious infection rate compared to adolescents (OR 2.76, 95% CI 1.99–3.83). The overall case-fatality rate was 0.98 per 100,000 infections without significant difference in age distributions.

**Table 2:** Estimates of age-specific rates of serious infections and fatalities among the general population with pandemic influenza A virus infections in Taiwan.

Character	Severe complicated cases			Fatal cases		
	No.	Case-serious infection rate(95% CI) per 100,000 infections	Odds ratio (95% CI)	No.	Case-fatality rate (95% CI)per 100,000 infections	Odds ratio (95% CI)
Age (years)						
<2	17	54.9 (28.8–81.0)	4.2 (2.5–7.2)	0	…	…
2–3	23	55.0 (32.6–77.5)	3.1 (1.8–5.3)	0	…	…
4–5	31	54.8 (35.5–74.0)	4.2 (2.8–6.5)	1	1.77 (0–5.23)	4.63 (0.42–51.06)
6–9	52	24.6 (17.9–31.3)	1.9 (1.3–2.7)	0	…	…
10–17	68	13.0 (9.9–16.1)	Referent	2	0.38 (0–0.91)	Referent
18–24	43	23.1 (16.2–30.0)	1.8 (1.2–2.6)	4	2.15 (0.04–4.25)	5.63 (0.97–30.73)
25–34	58	16.4 (12.2–20.6)	1.3 (0.9–1.8)	2	0.56 (0–1.35)	1.48 (0.21–10.50)
35–44	45	18.3 (13.0–23.7)	1.4 (0.97–2.1)	4	1.63 (0.03–3.23)	4.27 (0.78–23.34)
45–54	46	17.7 (12.6–22.8)	1.4 (0.9–2.0)	3	1.15 (0–2.45)	3.02 (0.50–18.07)
55–64	30	23.1 (14.9–31.4)	1.8 (1.2–2.7)	2	1.54 (0–3.68)	4.04 (0.57–28.69)
≥65	47	22.4 (16.0–28.8)	2.8 (2.0–3.8)	4	1.91 (0.04–3.78)	5.0 (0.92–27.30)
Overall	460	20.4 (18.6–22.3)		22	0.98 (0.57–1.39)	

### Factors associated with pH1N1 seropositivity in all participants

Seropositivity was significantly associated with age and the residing region of the subjects, but not with the other demographics (i.e., socioeconomic background including education of subjects and occupation of main incomer of family, family size, or underlying conditions; Table S1). Subjects living in Taipei had a lower seropositivity rate (26.1%) compared to those in Taoyuan (31.3%, p = 0.0524) or Tainan (33.3%, p = 0.0106). Compared to the general population, pregnant women appeared to have a lower seroprotection rate (30.4% vs. 20.0%, respectively) and GMT of HI (log titer, 1.43 vs. 1.42, respectively), but this did not reach statistical significance. Seropositivity was significantly higher for subjects with a history of influenza-like illness (ILI; p = 0.0308) and with vaccination against pH1N1 after June 2009 (p<0.0001). A higher rate was also evident in 52 subjects with laboratory evidence of pH1N1 infection (Table S1), including a positive result for the flu A rapid antigen test (p = 0.0028), hospitalization due to flu A (p = 0.0150) and ever on anti-flu medication (p = 0.0002). The significant factors associated with GMT of HI were similar to those in the seropositivity analysis except for region of sampling, for which the GMT of HI did not differ significantly (p = 0.1449).

Multivariate logistic regression analysis identified age <18 years (adjusted OR [aOR] 1.455, 95% CI 1.073–1.973, p = 0.0158), any laboratory evidence of pH1N1 infection (aOR 3.804, 95% CI 1.719–8.418, p = 0.0010), and immunization against pH1N1 (aOR 3.151, 95% CI 2.402–4.134, p<0.0001) as independent factors association with pH1N1 seropositivity. The geographic variables (Taoyuan vs. Taipei, p = 0.2826; Tainan vs. Taipei, p = 0.2421) and ILI (p = 0.3041) were not identified as significant factors in this analysis.

### Factors associated with pH1N1 seropositivity in unvaccinated participants

The analysis of epidemiological factors associated with seropositivity in unimmunized subjects is displayed in Table S2. Age and laboratory evidence of infection were the only two factors associated with pH1N1 seropositivity in the multivariate logistic regression analysis. Compared to the elderly (≥65 years old), children aged 6–9 years and adolescents aged 10–17 years had a 6.2- and 6.5-fold higher incidence of seropositivity (p = 0.0083 and 0.0003), respectively. With laboratory evidence suggesting pH1N1 infection was associated with a 3.8-fold higher rate of seropositivity.

## Discussion

Timely seroprevalence studies have been considered a more precise and reliable measure for estimating the extent of influenza infection in the community when compared to the surveillance systems based on clinical presentations (e.g., influenza-like illness) or laboratory confirmation, given that asymptomatic and most mild symptomatic patients do not seek medical attention [11]. However, most of the existing seroprevalence studies of the 2009 influenza A pandemic utilized convenient samples of residual serum from clinical laboratories or from blood donors, which could have resulted in under or over-estimates due to the selection bias [8], [12], [13], [14]. The pediatric population was frequently under-representative in these studies, which further compromised the precise estimate for the overall population. In the current study, the use of random samples from subjects at all ages should have allowed more accurate estimates of overall and age-specific seroprevalence of pH1N1 in the three studied regions of Taiwan. These areas resided a total of 10.3 million people, accounting for 44.9% of the whole population on the island. The seroepidemiology data before and after the pandemic along with the information for factors associated with post-pandemic seropositivity should be a useful reference for the national vaccine strategy and future public health interventions.

Results from the current study revealed a pH1N1 seroprevalence of approximately 30% in the general population after the 2009 pandemic, with the peak incidence in adolescents 10–17 years old and two declining trends toward the extremes of the age spectrum. The relatively low rate of seroprotection in young children and the elderly required further attention since they were the groups most vulnerable to severe influenza infections. As shown in the current study, young children 0–5 years of age with pH1N1 infections had greater than four–fold higher chance of developing severe diseases compared to adolescents 10–17 years of age. Greater disease severity was also identified in the elderly population. The finding was in contrast to the observation that young adults accounted for a majority of severely infected cases in the case-series studies [15], [16]. However, the observation of the case-series studies involving only severely infected cases should be interpreted cautiously. The greater number of serious infections occurring in young adults can be due to a higher pH1N1 attack rate in this population. In agreement with our analysis, a population-based study in Hong Kong clearly showed that, compared to children 5–14 years, elderly had 9.5 and 66 times higher risks of ICU admission and death, respectively, if infected with pH1N1. The severity of pH1N1 in young children (<6 years old) were less commonly reported in population-based study. Our data showed that young children had even greater morbidity once infected. A strategy to improve the uptake rate of the seasonal flu vaccines after pandemic would be needed in these two populations.

The finding that adolescents had the highest incidence of seropositivity could be the combined consequence of a high vaccine coverage rate and natural infection rate in this age group. According to the data from the Taiwan CDC, the age-specific vaccine uptake rate in the three regions of study was 29.2% (0–3 years), 19.7% (4–6 years), 62.0% (7–9 years), 71.4% (10–12 years), 74.7% (13–15 years), 63.1% (13–18 years), 3.1% (19–24 years), and 10.8% (>24 years) during the mass immunization campaign against pH1N1. The highest incidence of natural infections in schoolchildren and adolescents was also demonstrated by the distribution of seropositivity in the unimmunized population (Table 1) and further supported by a nation-wide virus surveillance network that disclosed a majority (55.9%) of pH1N1 isolates from adolescents and schoolchildren aged 6 to 15 years in 2009 epidemic (Taiwan CDC, unpublished data).

The extremely low incidence (0.91%) of subjects with pre-existing immunity against pH1N1 in Taiwan was unexpected. The data suggested very few Taiwanese had previous exposure to influenza A virus with antigenic similarity to pH1N1. The finding was in contrast to another small-scale study conducted in February and March of 2009 by our group, which indicated a seropositivity rate of 36.7% among 79 individuals aged ≥60 years [17]. The discrepancy may be due to the selection bias of the small-scale study, in which all of the study subjects were residents in a long-term care facility. The low overall seroprevalence of pH1N1 in the pre-pandemic period was also observed in selected populations in several Asian countries, including community-dwelling adults in Singapore (2.6%), farmers in rural southern China (1.7%), and children in a vaccine cohort study and adult blood donors in Hong-Kong (3.3%) [7], [18], [19]. In a Japanese study enrolling the workers, residents, and patients in health care facilities, seropositivity was rarely identified in subjects born after 1920 [20]. In contrast to Asian countries, the incidences of seropositivity were generally higher in western countries including Germany (13.1%), the United Kingdom (14.5–17.6%), the United States (6.0%), and New Zealand (11.9%) [11], [21]–[23]. A substantial proportion of individuals with pre-existing antibodies was commonly observed in the elderly (>60 years old) in these countries. The difference in seropositivity relative to geographic and age distribution is of great importance for the decision-making related to public health policy. For instance, the elderly was not among the priority groups in the mass immunization programs in most western countries [24], which may not be adequate in Asian countries considering the low incidence of pre-existing immunity and the greater disease severity of pandemic flu in this elderly population.

Except for natural infections and/or immunization, 6–17 years of age was the only epidemiological factor independently associated with increased incidence of seropositivity in unimmunized subjects. This data was in agreement with the finding in another seroprevalence study in New Zealand, which indicated children aged 5–19 years had the highest seropositivity rate and age was the most significant risk factor [23]. This observation suggested that children were at the greatest risk of infection during the global pandemic of the novel H1N1 influenza, which supported the idea that schoolchildren might be the main population accounting for pH1N1 transmission in the community and should be listed as the first priority for vaccination during the pandemic.

Due to the interference from the immunization program, it was difficult to precisely estimate the infection attack rate of pH1N1 in the general population based on serosurvey. In the current study, the estimate of severity was preceded using the data from unimmunized subjects based on the assumption that the seropositivity rate in the unimmunized population was proportional to whole population before immunization. Although convincing evidence supporting this assumption was lacking and the estimates of true infection numbers may be biased, we believed the relative severity of infection between each age group should be valid given that the sample subjects were randomly selected. The distribution of seropositivity among unimmunized subjects disclosed a relatively low seroprotection rate in young children and the elderly, suggesting a lower incidence of infections in these groups compared to others. This finding was in agreement with the seroepidemiology data before the implementation of the immunization program in several countries and areas [7], [8], [11], [19], [23]. With this estimate, we further observed a greater severity of pH1N1 infection in young children and the elderly than in adolescents and young adults, which was also consistent with the data from Hong Kong where the highest case-hospitalization rate occurred at the age of 5–14 years and 50–59 years (two most extreme age groups among the study subjects) [7]. The greater severity in young children and the elderly was also supported by a study estimating the case-fatality rate of pH1N1 infections in England [25].

There were several limitations in this seroprevalence study. First, the study was conducted 8 months after the second wave of the epidemic in Taiwan. Antibody decay to a level lower than the defined threshold may have occurred in some subjects. Recent studies further suggest that not all subjects developed seroprotection after pH1N1 infection and that the antibody level appears to be associated with the disease severity at presentation [26], [27]. We also observed that among subjects with laboratory evidence of pH1N1 infection, an HI titer greater than or equal to 1∶40 was identified in only 27 (51.9%) of 52 cases and in 6 (66.7%) of 9 hospitalized cases (Table 3). This observation suggested an underestimate of the true number of cases with natural infections, which inevitably led to the overestimation of the severity of infection. Second, some epidemiological factors influencing seropositivity may not have been collected. For instance, we have not collected the records of seasonal influenza vaccination, which was recently reported to have influence on the immunogenicity of pH1N1 infection or immunization [28], [29], [30]. Third, the study was conducted in three regions of Taiwan, and the seroprevalence data may not be generalized to the entire population of Taiwan. We noted that the seropositivity rate varied significantly from region to region. However, the impact of these geographic differences was controlled in the multivariate analysis of epidemiological risks. The significance of the identified factors associated with seropositivity should be valid in and applicable to the general population in Taiwan.

**Table 3:** Demographics of subjects with a determined antibody titer to novel influenza H1N1 in the pre-pandemic (2007) and post-pandemic (2010) periods.

Character	No. (%)	
	2007 (n = 1544)	2010 (n = 1558)
Age (years)		
<2	60 (3.9)	33 (2.1)
2–3	64 (4.2)	53 (3.4
4–5	56 (3.6)	137 (8.8)
6–9	84 (5.4)	70 (4.5)
10–17	150 (9.7)	121 (7.8)
18–24	159 (10.3)	155 (10.0)
25–34	195 (12.6)	237 (15.2)
35–44	196 (12.7)	218 (14.0)
45–54	199 (12.9)	217 (13.9)
55–64	158 (10.2)	168 (10.8)
≥65	223 (14.4)	149 (9.6)
Region		
Taipei (northern Taiwan)	586 (38.0)	591 (37.9)
Taoyuan (northern Taiwan)	489 (31.7)	517 (33.2)
Tainan (southern Taiwan)	469 (30.4)	450 (28.9)
Female gender	971 (62.9)	954 (61.5)

In summary, we observed an extremely low incidence (<1%) of pre-existing antibodies against pH1N1 in Taiwanese before the 2009 pandemic. The overall seroprotection rate increased to approximately 30% in the whole population and 20% in the unimmunized population after the pandemic, with significant variations among the different age groups. Adolescents had the highest incidence of seropositivity owing to the high rates of vaccine uptake and natural infections. Young children aged 0–5 years and elderly people aged ≥65 years were less likely to get infections during the 2009 pandemic; however, once infected, these individuals had up to 4.2 times higher risk of developing a serious complicated disease compared to adolescents at the age of 10–17 years. The finding of this seroepidemiology study enhanced our understanding of the influenza pandemic and could help shape the strategy of mass immunization for the future preparedness against pandemic flu. The data is also a useful reference for the public health authority in the coming flu season. For instance, boosting the immunity against pH1N1 in young children and the elderly via vaccination will be a priority in the winter of 2010 in Taiwan.

## Materials and Methods

### Ethics statement

A subset of serum samples collected in 2007 for other purpose (discussed in the next section) was used to estimate the seroprevalence of pH1N1 before the 2009 pandemic. The written informed consent to store and use the residual plasma was obtained from participants at the time of sample collection in 2007. The pre-pandemic serosurvey using residual serum samples was specifically reviewed and approved by the ethical committee in Chang Gung Memorial Hospital in July 2010. For the post-pandemic serosurvey, informed consent was obtained from all participants in 2010 and parental consent was further obtained for children younger than 18 years old. All informed consents were in written form. The post-pandemic serosurvey study was further reviewed and approved by the ethical committee in the Chang Gung Memorial Hospital in 2010.

### Study subjects

In 2007, a Taiwan CDC-funded survey was conducted to investigate the seroprevalence of several vaccine preventable diseases on the main island of Taiwan. A total of 3554 plasma samples were collected from the general population at all ages in four regions of Taiwan during August and October of 2007. The survey used a multi-stratified design to sample the civilians. In each region, age- and gender-stratified sampling was conducted using household registration records. A questionnaire-based interview was used to collect demographic data at the time of blood sampling. The plasma samples were frozen at −80°C following the seroprevalence survey of vaccine preventable diseases. In the current study, 1544 samples from subjects residing in three of the four regions (Taipei, Taoyuan, and Tainan) were selected for determination of antibody levels to the pH1N1. The sample set was used to estimate the seroprevalence of pH1N1 before the 2009 global pandemic in Taiwan.

To determine the post-pandemic seroprevalence of pH1N1, another 1558 serum samples from civilians residing in the same regions were obtained in September to October of 2010 using the same sampling strategy. The demographics including age, gender, residing region, occupation, education, and socioeconomics and information regarding underlying conditions (pregnancy, past history of influenza-like illness, influenza rapid test, flu medication, hospitalization due to pH1N1 in the past year, and vaccination against pH1N1) were obtained using a questionnaire-based interview. The laboratory evidences suggesting pH1N1 infection during pandemic included positive influenza rapid test, hospitalization due to flu A or use of anti-flu medication. The use of anti-flu medication was considered laboratory evidence of infection because of an official announcement in Taiwan asking for laboratory confirmation before prescribing anti-flu medication during the 2009 pandemic. The detailed age, region, and gender distributions of subjects in the 2007 and 2010 surveys are displayed in Table 3.

### Hemagglutination inhibition (HI) assay and microneutralization (MN) assay

Antibody responses to the 2009 pH1N1 (A/Taiwan/126/09) virus were detected by HI assay according to standard methods [31]. Each serum sample was treated with a receptor-destroying enzyme (RDE; Sigma-Aldrich, St. Louis, MO, USA) to inactivate nonspecific inhibitors. All samples were tested in duplicate at an initial dilution of 1∶10 and a final dilution of 1∶640. Positive control serum was obtained from a patient in Chang Gung Memorial Hospital with a laboratory-confirmed pH1N1 infection (RT-PCR positive) at the convalescent stage. Seropositivity and seroprotection were both designated as a HI titer ≥1∶40 to pH1N1. Geometric mean titer (GMT) was calculated for each age group, with a titer less than 1∶10 assigned a value of 5.

MN assay was performed in 336 (10.8% of all samples) randomly selected samples using an age-stratified sampling strategy and a web-based randomization service (http://www.random.org/lists/). The assay was done according to the protocol provided in World Health Organization Manual on Animal Influenza Diagnosis and Surveillance with minor modifications [32]. Each sample was tested in four repeats at an initial dilution of 1∶10 and a final dilution of 1∶1280. At the final step, the MDCK cells in wells were fixed with formalin and stained with crystal violet. The absorbance of the cells at 570 nm wavelength was measured spectophotometrically. A MN titer ≥1∶80 to pH1N1 was considered seropositive.

### Estimates of age-specific attack rates and severity of pH1N1

Mass vaccination campaign against pH1N1 in Taiwan was launched on 1 November, 2009. The populations among the priority groups of vaccination included health-care personnel, refugees of a strong summer storm in August 2009, pregnant women, children aged 6 months to 6 years and persons with catastrophic illness certificate issued by Bureau of National Health Insurance (http://nhi-test.pstcom.com.tw/english/webdata.asp?menu=11&menu_id=596&webdata_id=3180). Another nationwide in-school influenza vaccination programme targeting school-aged children (first to 12^th^ grade) was launched on 16 November, 2009 [33]. The remaining populations were encouraged to receive immunization since early December 2009. Until the end of January 2010, approximately 5,440,000 dosages of vaccine were administered and the uptake rate was estimated to be 23% in whole population.

The study subjects were first divided into two groups, either with or without vaccination against pH1N1. The occurrences of natural infection in vaccinees before immunization were unable to be estimated by the serosurvey. The seropositivity rate in unvaccinated subjects was therefore used as a proxy measure to estimate the infection attack rate in the general population. The rationale for this estimate was that the majority of the general population did not receive immunization during the major waves of the pH1N1 pandemic (Figure 1). Further, the study subjects were from random samples, and the probability of pH1N1 infection should have been similar among subjects with or without immunization before the mass vaccination program.

The age-stratified data for severe complicated infections and deaths associated with pH1N1 from July 2009 (the first severe case of pH1N1 infection in Taiwan) to Aug 2010 (before the serosurvey) were provided by the Taiwan CDC. The definition of severe complicated influenza included laboratory confirmation of pH1N1 by virus culture, RT-PCR, or seroconversion and at least one of the following clinical criteria: pulmonary complication requiring hospitalization, neurological complications, myocarditits or pericarditis, invasive bacterial infections, or other conditions requiring intensive care. The population statistics in the three sampling regions in 2009 were retrieved from the official website of the Department of Household Registration, Ministry of the Interior, Taiwan (http://www.ris.gov.tw/ch4/static/y0s109800.xls).

### Statistics

The comparison of categorical variables between subjects was performed with chi-square test or Fisher's exact test where appropriate. The difference between subjects on the numerical variables was tested using a two-sample *t-*test. The case-serious infection rates and case-fatality rates among different age groups was compared using Mantel-Haenszel method. Multiple logistic regression analysis was applied to explore factors associated with seropositivity. Statistical significance was defined as p<0.05. The data was analyzed with SAS software, version 9.1 (SAS Institute, Cary, NC, USA).

## Supporting Information

Table S1 **Demographics and epidemiological factors associated with seropositivity and geometric means of hemagglutination inhibition titers in 1558 Taiwanese after the 2009 H1N1 influenza pandemic.**
(DOCX)

Table S2 **Significant factors associated with seropositivity of pandemic influenza virus H1N1 in the subjects without immunization.**
(DOCX)
