# Supplementary material for: Seroprevalence and Severity of 2009 Pandemic Influenza A H1N1 in Taiwan
Source: PLoS One. 2011 Sep 1;6(9):e24440. doi: 10.1371/journal.pone.0024440 (PMC3164718; doi:10.1371/journal.pone.0024440)
Supplement: Table S1 — Demographics and epidemiological factors associated with seropositivity and geometric means of hemagglutination inhibition titers in 1558 Taiwanese after the 2009 H1N1 influenza pandemic. (DOCX) [file pone.0024440.s001.docx]

Table S1

| Character | Log of GMT  (mean±sd) | p | Seropositivity | | p |
| --- | --- | --- | --- | --- | --- |
|  |  |  | Rate (%) | OR (95% CI) |  |
| Gender |  | 0.1464 |  |  |  |
| Female (n = 954) | 1.42±0.25 |  | 28.7 | 0.872 (0.698-1.089) | 0.2270 |
| Male (n = 598) | 1.44±0.25 |  | 31.6 | Referent | - |
| Age |  | <0.0001 |  |  |  |
| <18 years (n = 414) | 1.48±0.32 |  | 38.9 | 1.751 (1.381-2.219) | <0.0001 |
| ≥18 years (n = 1144) | 1.41±0.23 |  | 26.7 | Referent | - |
| No. of family members |  | 0.3999 |  |  | 0.9886 |
| >4 persons (n = 532) | 1.44±0.28 |  | 29.9 | 0.998 (0.794-1.255) |  |
| ≤ 4 persons (n = 1026) | 1.42±0.25 |  | 29.9 | Referent |  |
| Region of Taiwan |  | 0.1449 |  |  |  |
| Taoyuan (n = 517) | 1.44±0.27 |  | 31.3 | 1.2949 (0.9972-1.6816) | 0.0524 |
| Tainan (n = 450) | 1.44±0.26 |  | 33.3 | 1.4188 (1.0846-1.8560) | 0.0106 |
| Taipei (n = 591) | 1.41±0.25 |  | 26.1 | Referent | - |
| Any underlying condition |  | 0.6819 |  |  |  |
| Yes (n = 164) | 1.42±0.23 |  | 29.3 | 0.966 (0.677-1.378) | 0.8495 |
| No (n = 1394) | 1.43±0.26 |  | 30.0 | Referent |  |
| Pregnancy after June 2009 |  | 0.0744 |  |  |  |
| Yes (n = 25) | 1.37±0.16 |  | 20.0 | 0.5713 (0.213-1.532) | 0.2603 |
| No (n = 1360) | 1.43±0.27 |  | 30.4 | Referent |  |
| Flu-like illness after June 2009 |  | 0.0128 |  |  |  |
| Yes (n = 204) | 1.48±0.29 |  | 36.3 | 1.405 (1.031-1.913) | 0.0308 |
| No (n = 1326) | 1.42±0.26 |  | 28.8 | Referent |  |
| Flu A rapid test after June 2009 |  | 0.030 |  |  |  |
| Positive (n = 18) | 1.62±0.35 |  | 61.1 | 3.8664(1.489-10.040) | 0.0028 |
| Negative or not checked(n = 1488) | 1.42±0.26 |  | 28.9 | Referent |  |
| Anti-flu drug after June 2009 |  | 0.0044 |  |  |  |
| Yes (n = 46) | 1.59±0.38 |  | 54.4 | 2.926 (1.621-5.284) | 0.0002 |
| No (n = 1485) | 1.42±0.25 |  | 28.9 | Referent |  |
| Hospitalization due to flu A after June 2009 |  | 0.0364 |  |  |  |
| Yes (n = 9) | 1.80±0.45 |  | 66.7 | 4.781 (1.190-19.198) | 0.0150 |
| No (n = 1527) | 1.42±0.26 |  | 29.5 | Referent |  |
| Laboratory evidences suggesting pH1N1 infection* |  |  |  |  |  |
| Yes (n = 52) | 1.57±0.38 | 0.0056 | 51.9 | 2.703 (1.551-4.714) | 0.0003 |
| No (n = 1452) | 1.42±0.25 |  | 28.5 | Referent |  |
| 2009 H1N1 immunization (any dose) |  | <0.0001 |  |  |  |
| Yes (n = 622) | 1.50±0.30 |  | 43.7 | 2.984 (2.382-3.739) | <.0001 |
| No (n = 912) | 1.38±0.22 |  | 20.6 | Referent |  |

sd, standard deviation

*Subjects with either a positive flu A rapid test, having received anti-flu medication, or hospitalization due to pandemic flu A after June 2009
