# Supplementary material for: Seroprevalence and Severity of 2009 Pandemic Influenza A H1N1 in Taiwan
Source: PLoS One. 2011 Sep 1;6(9):e24440. doi: 10.1371/journal.pone.0024440 (PMC3164718; doi:10.1371/journal.pone.0024440)
Supplement: Table S2 — Significant factors associated with seropositivity of pandemic influenza virus H1N1 in the subjects without immunization. (DOCX) [file pone.0024440.s002.docx]

Table S2.

| Character | H1N1 seropositivity | | | | | |
| --- | --- | --- | --- | --- | --- | --- |
|  | Rate (%) | Univariate analysis | |  | Multivariate analysis | |
|  |  | OR (95% CI) | p |  | Adjusted OR (95% CI) | p |
| Age |  |  |  |  |  |  |
| <2 (n = 23) | 17.4 | 1.569 (0.450-5.469) | 0.4784 |  | 1.408 (0.391-5.061) | 0.4193 |
| 2-3 (n = 17) | 23.5 | 2.294 (0.634-8.293) | 0.1982 |  | 2.083 (0.543-7.989) | 0.9638 |
| 4-5 (n = 67) | 28.4 | 2.951 (1.295-6.724) | 0.0084 |  | 2.137 (0.864-5.284) | 0.9975 |
| 6-9 (n = 23) | 47.8 | 6.833 (2.434-19.183) | <0.0001 |  | 6.178 (2.121-17.995) | 0.0083 |
| 10-17 (n = 44) | 47.3 | 6.806 (2.870-16.144) | <0.0001 |  | 6.518 (2.624-16.187) | 0.0003 |
| 18-24 (n = 117) | 22.2 | 2.130 (0.991-4.580) | 0.0501 |  | 2.183 (0.981-4.854) | 0.9244 |
| 25-34 (n = 148) | 18.9 | 1.739 (0.820-3.689) | 0.1465 |  | 1.931 (0.885-4.214) | 0.6498 |
| 35-44 (n = 139) | 20.1 | 1.880 (0.885-3.995) | 0.0976 |  | 1.946 (0.887-4.272) | 0.6819 |
| 45-54 (n = 137) | 14.6 | 1.274 (0.80-2.802) | 0.5468 |  | 1.316 (0.579-2.991) | 0.0546 |
| 55-64 (n = 104) | 15.4 | 1.355 (0.594-3.091) | 0.4698 |  | 1.544 (0.658-3.623) | 0.2370 |
| ≥65 (n = 93) | 11.8 | Referent |  |  | Referent |  |
| Region of Taiwan |  |  |  |  |  |  |
| Taoyuan (n = 231) | 22.9 | 1.497 (1.025-2.188) | 0.0364 |  | 1.213 (0.780-1.886) | 0.3914 |
| Tainan (n = 274) | 23.7 | 1.434 (0.961-2.139) | 0.0773 |  | 1.471 (0.980-2.207) | 0.0623 |
| Taipei (n = 407) | 17.2 | Referent |  |  | Referent |  |
| Flu-like illness after June 2009 |  |  |  |  |  |  |
| Yes (n = 109) | 30.3 | 1.828 (1.171-2.854) | 0.0072 |  | 1.306 (0.752-2.270) | 0.3437 |
| No (n = 792) | 19.2 | Referent |  |  | Referent |  |
| Flu A rapid test |  |  |  |  |  |  |
| Positive (n = 10) | 70.0 | 9.630 (2.465-37.621) | <0.0001 |  |  |  |
| Negative or not checked (n = 887) | 19.5 | Referent |  |  |  |  |
| Anti-flu drug |  |  |  |  |  |  |
| Yes (n = 24) | 58.3 | 5.713 (2.495-13.078) | <0.0001 |  |  |  |
| No (n = 884) | 19.7 | Referent |  |  |  |  |
| Hospitalization due to flu A after June 2009 |  |  |  |  |  |  |
| Yes (n = 5) | 80.0 | 15.781 (1.753-142.044) | 0.0010 |  |  |  |
| No (n = 905) | 20.2 | Referent |  |  |  |  |
| Laboratory evidence suggesting pH1N1 infection |  |  |  |  |  |  |
| Yes (n = 26) | 57.7 | 5.689 (2.567-12.609) | <0.0001 |  | 3.791 (1.327-10.829) | 0.0128 |
| No (n = 874) | 19.3 | Referent |  |  | Referent |  |
